# Analysis of Strain Hardening Processes of AISI 316 LN Austenitic Stainless Steel

**DOI:** 10.3390/ma18184268

**Published:** 2025-09-12

**Authors:** Tibor Kvačkaj, Jana Bidulská, Ľuboš Kaščák, Alica Fedoríková, Róbert Bidulský

**Affiliations:** 1 Bodva Industry and Innovation Cluster, Budulov 174, 04501 Moldava and Bodvou, Slovakia; director@biic.sk; 2 Department of Metallic Materials, Institute of Materials, Faculty of Materials, Metallurgy and Recycling, Technical University of Kosice, Park Komenského 11, 04200 Košice, Slovakia; 3 Department of Technology, Materials and Computer-Aided Production, Faculty of Mechanical Engineering, Technical University of Košice, Letná 9, 04002 Košice, Slovakia; lubos.kascak@tuke.sk; 4 Department of Material Analysis, Research Centre Řež, Hlavní 130, 25068 Husinec, Czech Republic

**Keywords:** AISI 316 LN, cold rolling, mechanical properties, strain hardening rate, strain hardening coefficient, recovery rate

## Abstract

The primary objective of this contribution is to numerically and graphically evaluate engineering stress–strain curves, transform them into true stress–strain curves, and de-scribe the key points of material processed by cold rolling with strains of ε_Roll_ = 0%, 10%, 30%, and 50%. The initial and final conditions for uniform plastic deformations have been described. The initial point of uniform deformation lies above the onset yield strength value (σ_T,S_ > R_P0,2_). The necking point, as the final point of uniform deformation, was determined as the intersection point of the curves of the true stress–strain and strain hardening rate. The strain hardening coefficient and the recovery rate, as a function of cold rolling deformations, were derived. Convex polyhedra were derived which describe the dependencies of the development of maximal strain hardening rate values (θ_Max_) and initial strain hardening rates (θ_0_) as a function of cold rolling deformations and the diameter of grain. The decisive point at which the curves showed a local maximum was a cold rolling deformation ε_Roll_ = 30%. The saturation stress required to initiate dynamic recovery of the microstructure is significantly higher than the stress necessary for necking (σ_T,Sat_ > σ_T,Neck_). The saturation strain required to initiate dynamic recovery of the microstructure is significantly higher than the strain needed for necking formation (ε_T,Sat_ > ε_T,Neck_).

## 1. Introduction

The strength properties of metallic materials depend on which passive and active strengthening contributions have been made to them. Passive strengthening contributions are primarily determined by the chemical composition, which depends on the content of interstitial and substitutional elements, as well as their potential mutual chemical interactions. Additionally, they are influenced by the lattice friction stress, also known as the resistance force to dislocation movement (Peierls–Nabarro stress). More important from the point of view of the strengthening gradient are the active strengthening contributions, which are predominantly dependent on the deformation conditions, such as grain boundaries, deformation twins, and dislocation density, often referred to by the common term strain hardening parameters [1]. Strain hardening is a widely used technique for achieving high-strength properties in metallic materials. The strength properties of austenitic stainless steel (SS) are primarily a function of the face-centred cubic (FCC) crystallographic system of austenite [2,3] and its ability to initiate dynamic recovery and dynamic recrystallisation, which depends on the thermo-deformation conditions [4,5].

A graphical representation characterising the relationship between stress and strain can be described using stress–strain curves, which result from static tensile tests of the observed material.

For description, the strain hardening rate (θ = dσ/dε) is important, as it represents the part of the stress–strain curve where the deformation simultaneously increases with increasing stress. This part of the curve is known as uniform deformation, which occurs after the yield point has been reached. Uniform deformation is terminated at the singularity point, where strain hardening changes to geometric softening. After reaching the singular point, the following inequality holds: (dσ/dε) < 0 [6]. According to the authors [7], the onset of plastic instability is accompanied by the formation of the necking, which can be represented by formula θ = dσ/dε)/σ ≈ 0. At this point, the material transitions from the stage of plastic stability to the stage of plastic instability, also known as the point at which necking begins. This means that the plastic instability of a material can be achieved when the curve of the strain hardening rate crosses the curve of the true stress–strain [7].

The numerical description of stress–strain curves, carried out by authors Voce, Swift, Hollomon, Ludwig, Pickering, Crussard and Jaoul, and Bergstrom using strain hardening models, is presented in works [8,9,10,11]. The simplest model for describing true stress–strain curves is the Hollomon equation:

(1) $$\sigma \left(\varepsilon \right)=K\cdot \varepsilon^{n}$$

where:

σ(ε) [MPa]—flow stress;

ε [-]—strain;

K [MPa]—strength coefficient;

n [-]—strain hardening exponent;

ε_U_≡ε_Neck_ [-]—true strain at which the beginning of the necking occurs.

The strain hardening exponent differs from the strain hardening rate, and is described as the first derivative of the function (θ = dσ(ε)/dε) [12,13,14]. The relationship for the strain hardening coefficient can be expressed as follows:

(2) $$n=\frac{d\left(\ln\sigma \left(\varepsilon \right)\right)}{d\left(\ln\left(\varepsilon \right)\right)}=\frac{\varepsilon }{\sigma }\cdot \frac{d\left(\sigma \left(\varepsilon \right)\right)}{d\left(\varepsilon \right)}=\varepsilon_{U}$$

Very useful parameters that describe the onset of homogeneous (uniform) deformation on a stress–strain curve are the strain hardening rate and the strain hardening coefficient. Both parameters can only be evaluated when the material underloading exceeds the yield point value.

The peak strain and peak stress (ε_Peak_;σ_Peak_) are determined at the point with θ = dσ/dε = 0, which indicates that a balance between strain hardening and softening is reached. The saturation strain and saturation stress (ε_Sat_; σ_Sat_) are defined as the extrapolation of the dependence θ = f(ε;σ) to the value θ = 0. In other words, it is the point where the extrapolation curve θ = f(ε;σ) intersects the ε-axis and σ-axis [15]. The saturation stress (σ_Sat_) represents a dynamic equilibrium between strain hardening and recovery, where a decrease in the dislocation density occurs due to the rearrangement and annihilation of dislocations through slip, climb, and sub-boundary migration [11,16]. The initial strain hardening rate (θ_0_) is determined as the point where the extrapolation curve θ = f(σ) intersects the σ-axis. The maximum strain hardening rate (θ_Max_) is defined as the point where the extrapolation curve θ = f(σ) intersects the θ-axis [17].

This study focuses on investigating stress–strain curves resulting from cold rolling deformations to describe and analyse them numerically. The relationships between strain hardening rates and true stress, as well as the relationships between the true stress and the strain hardening exponent and recovery rate of the steel grade AISI 316LN, are discussed for an FCC polycrystalline metal.

## 2. Materials and Experimental Procedure

Austenitic AISI 316 LN stainless steel with the chemical composition given in Table 1 was studied. Chemical composition results were obtained from spectroscopic measurements.

The cast ingot was hot forged into a flat shape, from which samples for cold rolling with dimensions h0 × b0 × l0 = 15 × 40 × 75 mm were taken. Before cold rolling, the samples were annealed at 777 K for 60 min, which was followed by rapid air cooling to ambient temperature. 

Cold rolling experiments were performed at room temperature (295 K) on duo rolling mill with roll diameter of 210 mm and rolling rate of 1 m/min. The thickness reduction per each pass was 10%. Specimens for static tensile tests were machined from cold-rolled samples with different total thickness reductions of 10%, 30% and 50%. The shape of the specimens for static tensile tests was circular with a diameter and length of the measurement part of d = 4 mm and l = 22 mm. The axes of the specimens for static tensile tests were parallel to the rolling direction. Each point representing mechanical properties is the average value from three specimens after static tensile tests. The displacement rate of the static tensile tests was 0.5 mm/min. Specimen dimensions and static tensile tests were performed in accordance with ASTM E8M [18]. 

## 3. Results and Analysis

### 3.1. Stress-Strain Curves

**Figure 1:**
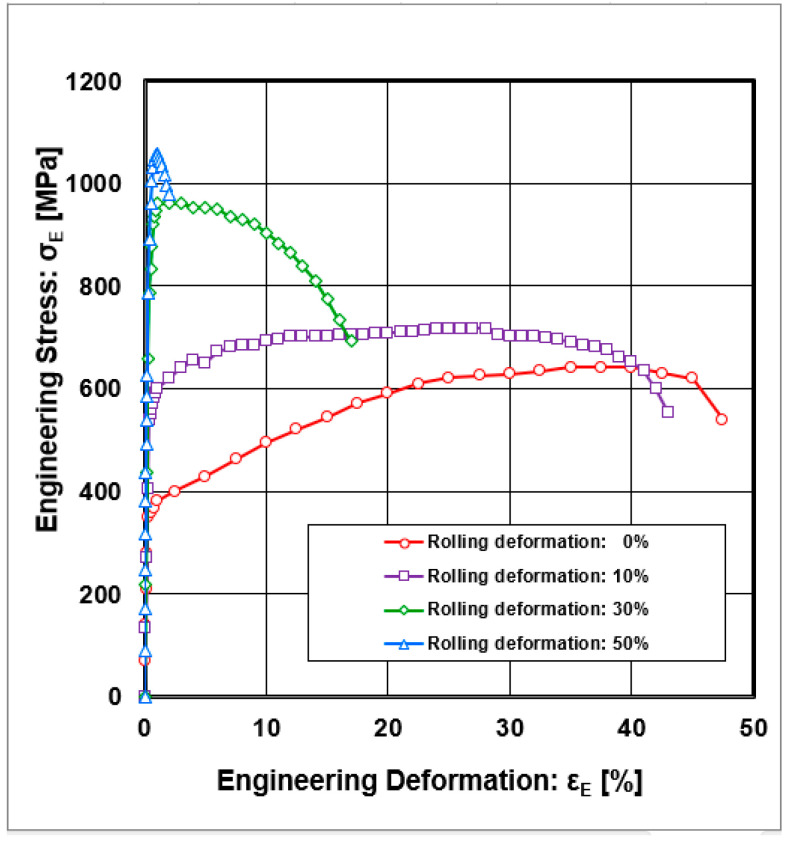
Engineering stress–strain curves σ_E_ = f(ε_E_) after static tensile tests at 295 K.

The stress–stain curves resulting from static tensile tests performed at 295 K on cold-rolled samples with varying thickness reductions (0%, 10%, 30%, 50%) are shown in Figure 1.

**Figure 2:**
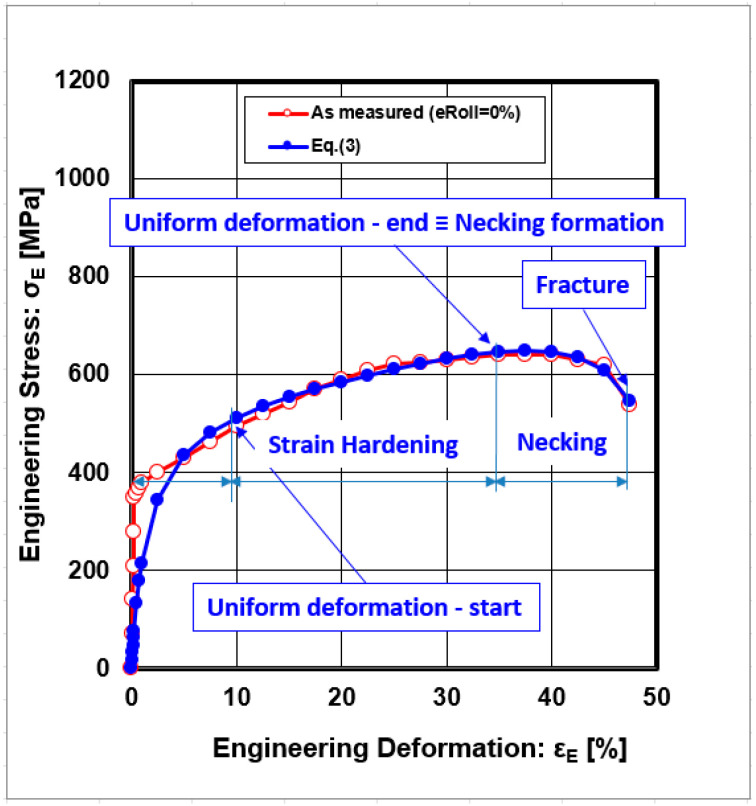
The basic points characterised for stress–strain curves (ε_Roll_ = 0%).

A graphical representation of the relationship between stress and strain resulting from static tensile tests of a material sample, known as the engineering stress–strain dependence, with definitions of the essential points for describing the stress–strain curve in terms of the necessary information for deformation strengthening, is shown in Figure 2.

Important points that describe the curves of the strain hardening rate are the point where uniform deformation begins and the point where uniform deformation ends (also called the necking point on the tested specimen). The last point is the fracture of the sample.

The numerical description of the curves σ_E_ = f(ε_E_), representing the measured data, was performed using the following equations:

(3) $$\sigma_{E}=\left(298\cdot \varepsilon_{E}-5.8\cdot \varepsilon_{E}^{2}\right)/\left(1+0.475\cdot \varepsilon_{E}-0.012\cdot \varepsilon_{E}^{2}+0.000045\cdot \varepsilon_{E}^{3}\right)\;$$

where:

σ_E_ [MPa]—engineering stress;

ε_E_ [-]—engineering strain;

R^2^ = 0.91.

The graphical dependencies shown in Figure 2 yield a suitable approximation of the measured values by the regression Equation (3), in the interval from the start to the end of strain hardening.

Transformation of the engineering stress–strain curves to true stress–strain curves was carried out according to the following formulae [19,20]:

(4) $$\sigma_{T}=\sigma_{E}\cdot \left(1+\varepsilon_{E}\right)$$

(5) $$\varepsilon_{T}=\ln\left(1+\varepsilon_{E}\right)$$

For the approximation of the calculated values, σ_E_ = f(ε_T_) was used in the following formulae:

(i) A approximate regression equation was derived in the following form:
  (6) $$\sigma_{T}=\left(A\cdot \varepsilon_{T}+B\cdot \varepsilon_{T}^{2}\right)/\left(1+C\cdot \varepsilon_{T}+D\cdot \varepsilon_{T}^{2}+E\cdot \varepsilon_{T}^{3}\right)$$

Regression coefficients for Equation (6) are given in Table 2.

(ii) Holloman equation (Equation (7)):
  (7) $$\sigma_{T}=K\cdot \varepsilon_{T}^{n}$$

Calculated coefficients for Equation (7) are given in Table 3.

**Figure 3:**
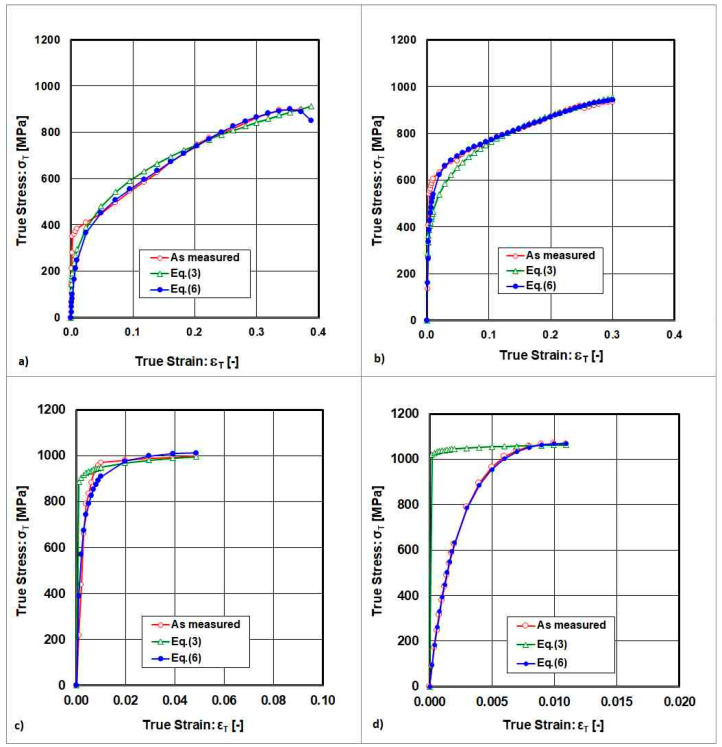
True stress–strain curves resulting from measurement and approximation by Equations (6) and (7). (**a**) State after solution annealing, (**b**) state after cold rolling deformation ε_Roll_ = 10%, (**c**) state after cold rolling deformation ε_Roll_ = 30%, (**d**) state after cold rolling deformation ε_Roll_ = 50%.

The transformation of engineering stress–engineering strain curves into true stress–true strain curves with the application of Equations (6) and (7) is shown in Figure 3.

#### 3.1.1. The Manner of Determination of Important Points

The starting point of uniform deformation described by the coordinates (ε_T,S_;σ_T,S_) was graphically determined from the following function dependence:

(8) $$\theta =\frac{d\sigma_{T}}{d\varepsilon_{T}}=f\left(\varepsilon_{T}{;\sigma }_{T}\right)$$

where:

θ [MPa]—the strain hardening rate calculated as the first derivative of true stress according to the true strain.

**Figure 4:**
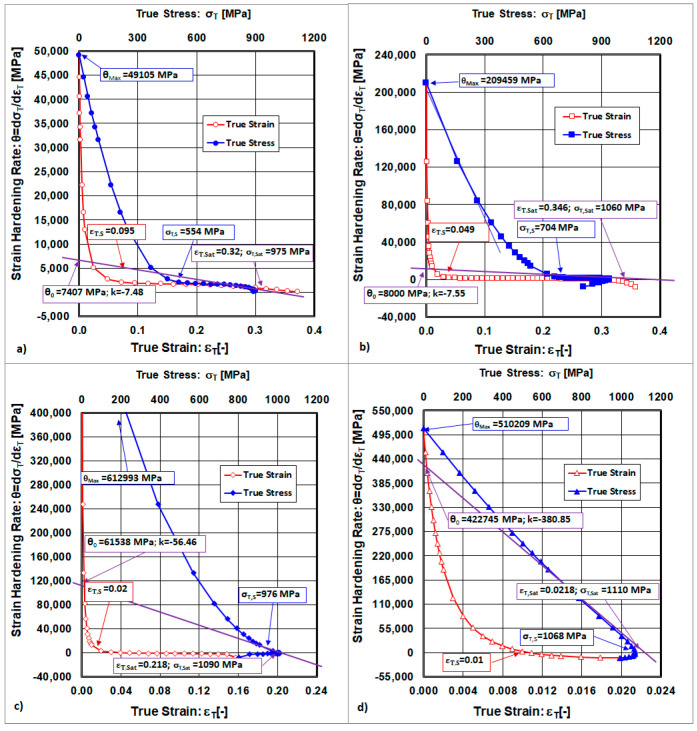
Basic points resulting from curves. (**a**) State after solution annealing, (**b**) state after cold rolling deformation ε_Roll_ = 10%, (**c**) state after cold rolling deformation ε_Roll_ = 30%, (**d**) state after cold rolling deformation ε_Roll_ = 50%.

The graphical representations of Equation (8) are shown in Figure 4.

The point where the tangent to the curve θ = f(σ_T_) intersects the axis θ = dσ_T_/dε_T_ was characterized by the authors of [17,21,22] as the initial strain hardening rate (θ_0_). The point where the curve θ = f(σ_T_) intersects the axis θ = dσ_T_/dε_T_ was classified by the authors as the maximal strain hardening rate (θ_Max_). The relationship between the initial strain hardening rate (θ_0_) and the saturation stress (σ_T,Sat_) shown in Figure 4 is then described as the recovery rate (k [-]) by the following formula:

(9) $$k=\theta_{0}/\sigma_{T,Sat}$$

Determination of saturated points described by coordinates (ε_T,Sat_; σ_T,Sat_) is given as the extrapolation of the curve θ = f(σ_T_) intersecting the ε_T_-axis and σ_T_-axis when θ = 0, as shown in Figure 4. The saturation stress (σ_T,Sat_) represents a dynamic equilibrium between the strain hardening and the recovery. An increase in cold rolling deformation leads to an increase in the dislocation density, while a decrease in the dislocation density occurs because of the rearrangement and annihilation of dislocations by slip [11].

**Figure 5:**
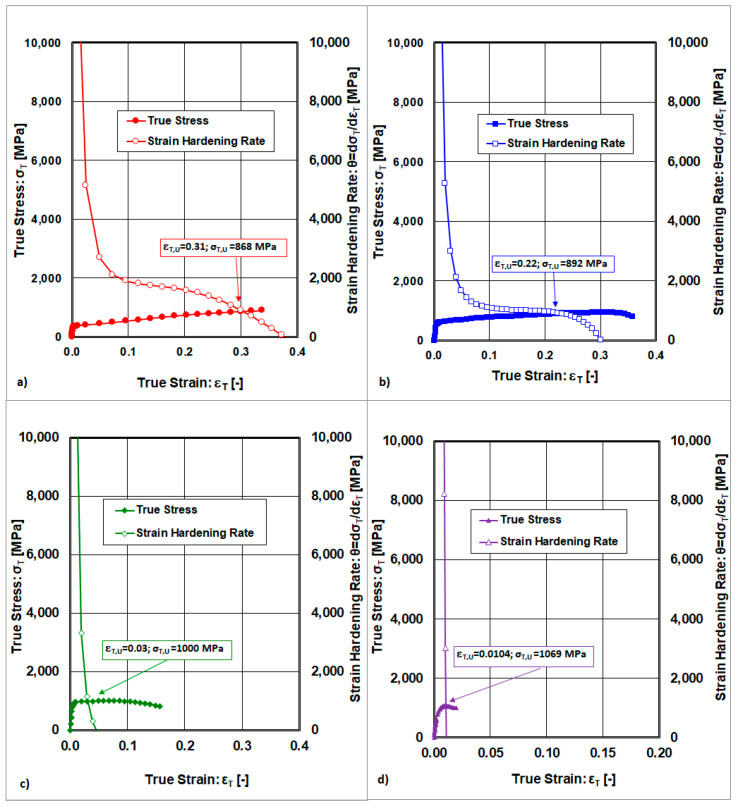
The manner of determining the starting point of the necking: (**a**) state after solution annealing, (**b**) state after cold rolling deformation ε_Roll_ = 10%, (**c**) state after cold rolling deformation ε_Roll_ = 30%, (**d**) state after cold rolling deformation ε_Roll_ = 50%.

The final point at which uniform deformation is terminated (also called the necking point), described by the coordinates (ε_T,U_≡ε_T,Neck_; σ_T,U_≡σ_T,Neck_), is determined as the intersection point of the two curves corresponding to the onset of necking. One curve describes the true stress–strain curve and the other curve characterises the strain hardening rate, and both depend on the true strain, as shown in Figure 5. The intersection point of the two curves corresponds to the onset of necking. It is assumed that determining the necking onset directly from a static tensile test is more inaccurate than determining it from the described method.

**Figure 6:**
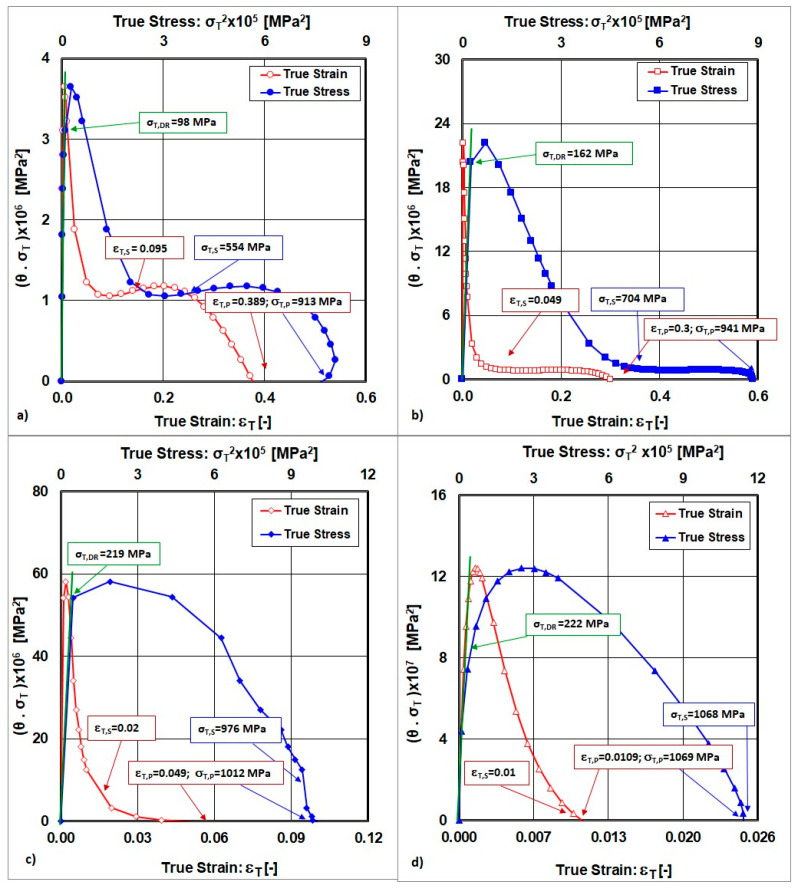
The manner of determining the peak point and dynamic recovery true stress. (**a**) state after solution annealing, (**b**) state after cold rolling deformation ε_Roll_ = 10%, (**c**) state after cold rolling deformation ε_Roll_ = 30%, (**d**) state after cold rolling deformation ε_Roll_ = 50%.

The determination of the peak point described by the coordinates (ε_T,P_≡ε_T,Peak_; σ_T,P_≡σ_T,Peak_) is achieved under the condition that the product θ.σ_T_ = 0, which indicates that a balance between strain hardening and softening was reached, as is shown in Figure 6.

The dynamic recovery true stress (σ_T,DR_) is determined as a tangent to the curves for θ.σ_T_ = f(σ_T_^2^), which are given in Figure 6. Dynamic recovery can occur at a low deformation temperature and higher strain rate in materials with low to medium values for their stacking fault energy, such as AISI 316LN-grade steel [1,16,23].

The determination of the strain hardening exponent (n) is based on Equation (2) and Figure 5, where it is valid that n = ε_T,U_.

The coefficient (K) in the Hollomon equation (Equation (7)) is determined as the intersection of the extrapolated curve ln(σ_T_) = f(ε_T_ = 1), with a vertical line being erected at the point (ε_T_ = 1). “K” represents the true stress at ε_T_ = 1.

#### 3.1.2. Analysis of Dependencies

(a) Relationships between strain hardening rates and true stress

The common relationship between strain hardening rates and true stresses, described by the dependencies θ_0_ = f(σ_T,U ≡ Neck_), θ_0_ = f(σ_T,S ≡_ σ_T,Start)_, θ_0_ = f(σ_T,Peak_), θ_0_ = f(σ_T,DR_), θ_Max_ = f(σ_T, U ≡ Neck_), θ_Max_ = f(σ_T, S ≡_ σ_T,Start_), θ_Max_ = f(σ_T, Peak_), and θ_Max_ = f(σ_T,DR_), will be analysed.

The mathematical dependencies describing the relationship between strain hardening rates (θ_Max_, θ_0_) and true stresses are given in Table 4.

**Figure 7:**
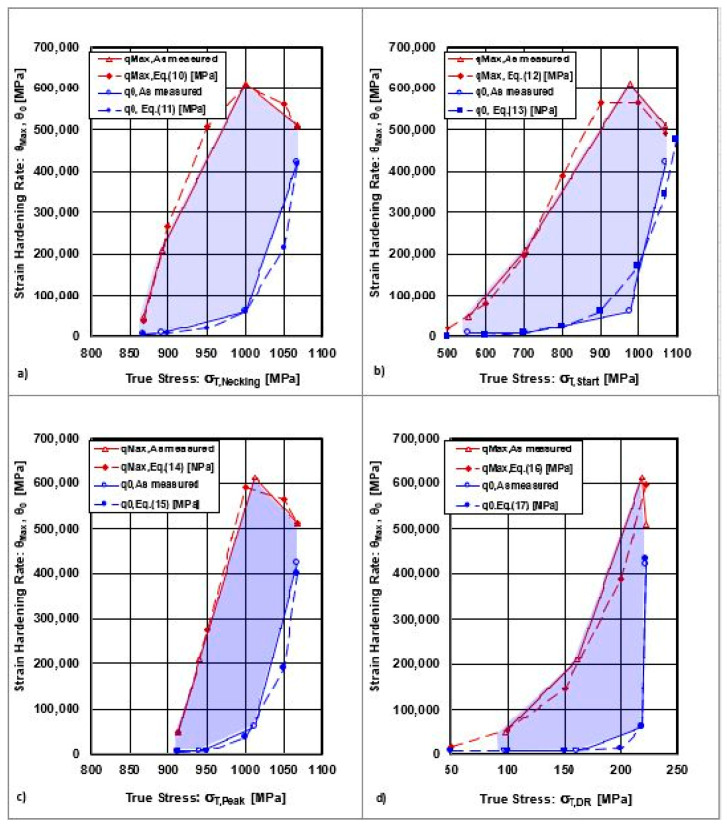
Dependences of the strain hardening rates on uniform stresses. (**a**) state after solution annealing, (**b**) state after cold rolling deformation ε_Roll_ = 10%, (**c**) state after cold rolling deformation ε_Roll_ = 30%, (**d**) state after cold rolling deformation ε_Roll_ = 50%.

The graphical interpretations of the previous equations are shown in Figure 7.

**Figure 8:**
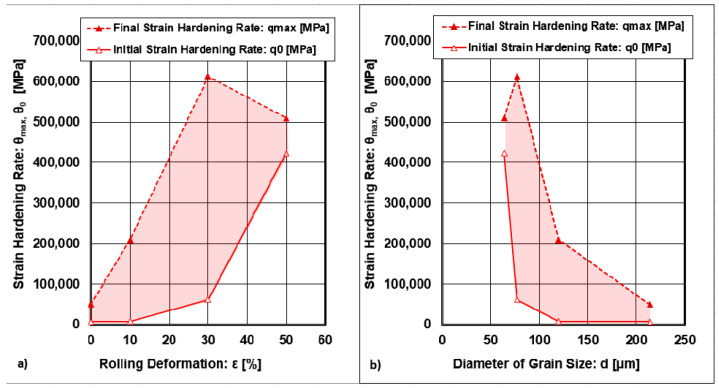
Dependence of strain hardening rates on and diameter of grain. (**a**) cold rolling deformations; (**b**) diameter of grain size.

The graphical dependencies of q_Max_=f(s_T_) in Figure 7 show that the value of the maximal strain hardening rates reaches one local maximum. The curve showed an increasing tendency until reaching a local maximum. On the other hand, the curve θ_0_ = f(σ_T_) exhibits an exponential dependence without a local maximum. While the curve θ_0_ represents the set of points for the initial strain hardening rates, the curve θ_Max_ represents the set of points for the final strain hardening rates. Both dependencies shown in Figure 7 and Figure 8 indicate the minimum and maximum boundaries of a convex polyhedron, within which there may be points representing nonoptimal conditions of plastic deformation. The optimal points lie on the curves. The dependencies of strain hardening rates on cold rolling deformations are shown in Figure 8.

The local maximum of the function θ_Max_ = f(ε_Roll_) was reached at the point (ε_Roll_;θ_Max_) = (30%; 612,993 MPa). Figure 8 shows that all local maxima correspond to cold rolling deformation ε_Roll_ = 30%. This change in θ_Max_ after ε_Roll_ = 30% cold deformation is related to the decrease in dislocation density, which was discussed in detail in previous work [1]. The curves for θ_Max_,θ_0_ = f(d) are represented by the opposite dependence, where the strain hardening rates decrease with an increasing grain size. The curve θ_Max_ = f(d) exhibits a local maximum at the point where the diameter of the grain size d = 77 μm, which also corresponds to ε_Roll_ = 30%.

(b) Relationships between true stress and strain hardening exponent and recovery rate

The relationship between the true stresses and strain hardening exponent and the recovery rate is described by the dependencies σ_T,Sat_ = f(n;k), σ_T,DR_ = f(n;k), and n = f(k), and will be analysed.

The mathematical dependencies describing the relationship between the true stresses (σ_T,Sat_, σ_T,DR_) and strain hardening exponent (n) and the recovery rate (k) are given in Table 5.

**Figure 9:**
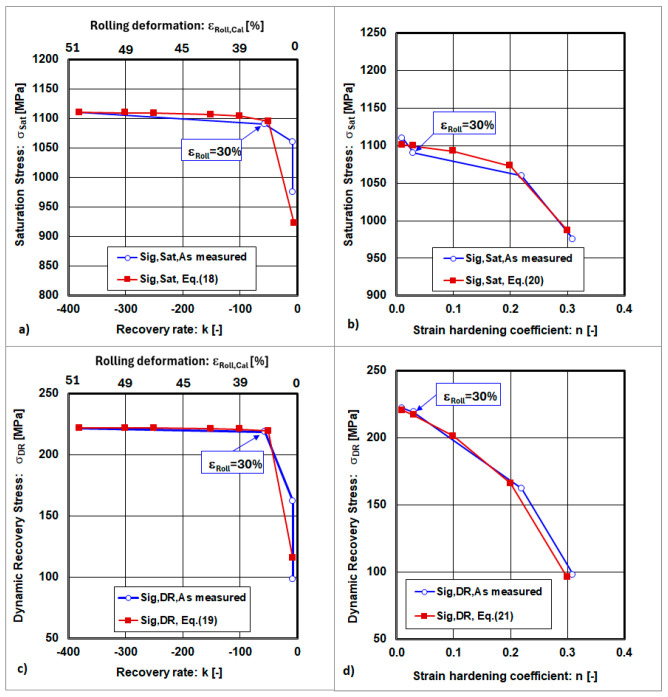
Dependences of the saturation stress and dynamic recovery stress on the recovery rate and strain hardening coefficient. (**a**) state after solution annealing, (**b**) state after cold rolling deformation ε_Roll_ = 10%, (**c**) state after cold rolling deformation ε_Roll_ = 30%, (**d**) state after cold rolling deformation ε_Roll_ = 50%.

**Figure 10:**
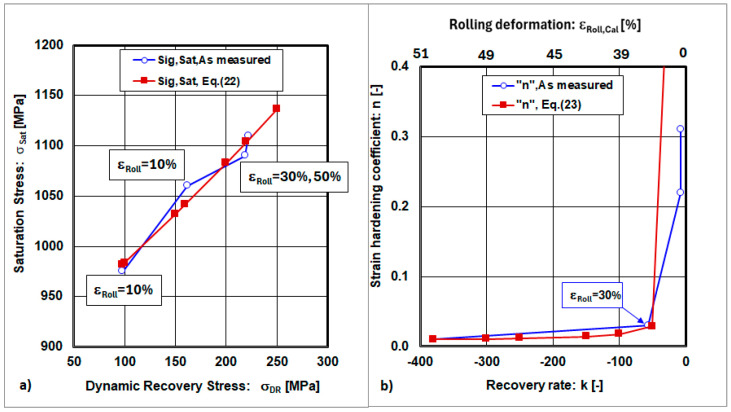
Dependences: (**a**) saturation stress on dynamic recovery stress, (**b**) strain hardening coefficient on the recovery rate.

The graphical interpretations of the previous equations are shown in Figure 9 and Figure 10.

The relationships between σ_Sat_, σ_DR_, and n = f(k), which depend on the recovery rate (k), exhibit local extrema at the point where the cold rolling deformation has reached a value of ε_Roll_ = 30.

**Figure 11:**
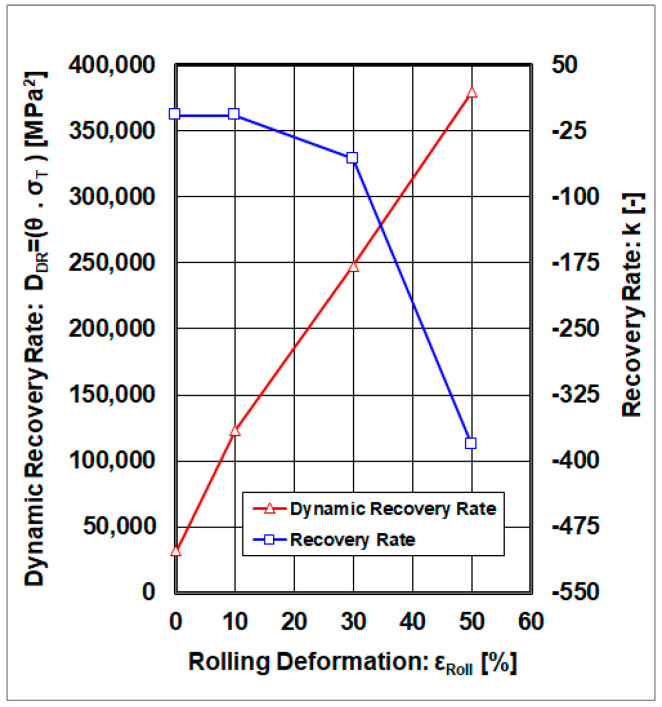
Dependence of the dynamic recovery rate and the recovery rate on cold rolling deformations.

The graphical dependencies of the dynamic recovery rate and the recovery rate, as a function of cold rolling deformations, are shown in Figure 11. The graphical interpretation indicates that the dynamic recovery rate increases with increasing cold rolling deformations. This means that, with increasing cold plastic deformation, the accumulated energy, which is jointly dependent on the dislocation density and deformation twinning, increases and the ability of recovery rate processes is reduced. The accumulated energy, as described by the dynamic recovery rate (DDR), is not sensitive to cold rolling deformations. On the other hand, the recovery rate (k) exhibits a dependence on cold rolling deformations, with a local extreme at the point ε_Roll_ = 30%.

**Figure 12:**
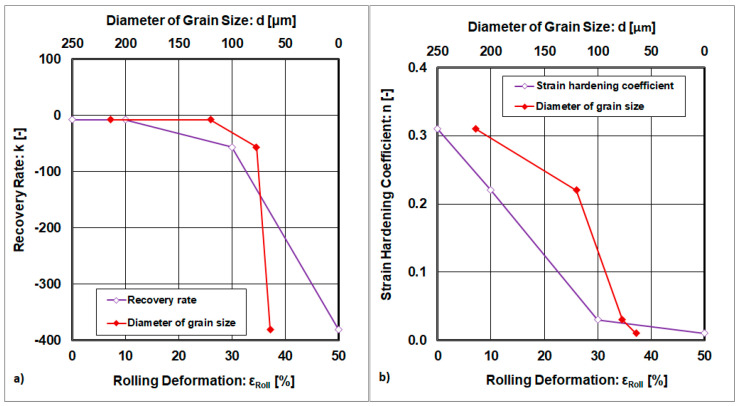
Dependences of (**a**) the recovery rate and strain hardening exponent on processing (ε_Roll_) and microstructure (**b**) parameters.

Relationships based on dependence k and n = f(ε_Roll_; d), i.e., on the cold plastic deformation and diameter of the grain, are given in Figure 12.

The recovery rate falls with increasing cold rolling deformation and declines with the grain size as is shown in Figure 12a. Similar dependencies are also evident in the strain hardening exponent, but without any significant local extremes as is given in Figure 12b. The declining dependencies are based on microstructural refinement and changes in the mechanisms of plastic deformation. At the point ε_Roll_ = 30%, the transformation of low-angle grain boundaries to high-angle grain boundaries begins.

**Figure 13:**
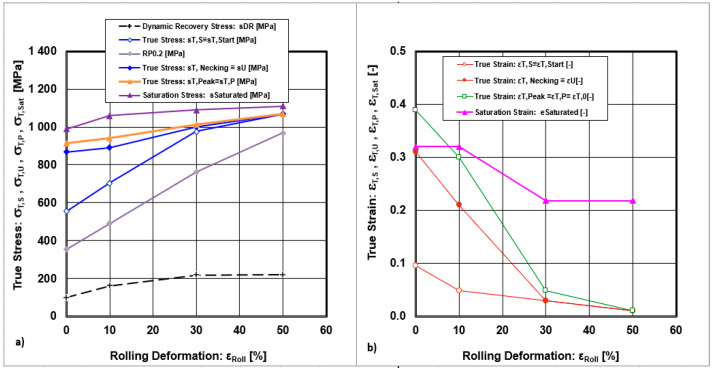
Comprehensive analysis describing dependencies: (**a**) true stresses on cold rolling deformations, (**b**) true strains on cold rolling deformations.

A comprehensive analysis of the previous graphical dependencies, based on the assessment of true stresses and true strains in relation to cold rolling deformations, is presented in Figure 13.

The curves describing the dependences of the true stress for the starting of uniform plastic deformation (σ_T,S_) and the true stress for the finishing of uniform plastic deformation (σ_T,Neck_) point to the fact that, with increasing cold deformation, the area of the convex polyhedron for possible values of uniform stresses decreases significantly. The stress convex polyhedron of uniform plastic deformations is located above the curve describing the offset yield strength (R_P0.2_). The graphical dependences of the stresses indicate that the saturation stress (σ_T,Sat_) required to achieve dynamic recovery of the microstructure is significantly higher than the stress required for necking formation (σ_T,Sat_ > σ_T,Neck_). This fact is also confirmed by the decreasing recovery rate shown in Figure 12a. Analysis of the material studied reveals that the dynamic recovery of the microstructure under cold plastic deformation conditions is limited. The true peak stresses are very similar to true necking stresses (σ_T,Peak_ ≈ σ_T,Neck_).

The convex polyhedron of the set of true strains is limited by the curves of the true strain of uniform plastic deformation (ε_T,S_) and the true strain finish of uniform plastic deformation (ε_T,Neck_). The true strain values decrease with increasing cold rolling deformation. Also, the interval describing the set of points of the true strains decreases significantly with increasing cold deformation. After reaching a cold deformation of ε_Roll_ ≥ 30%, its complete elimination occurred. The values of true saturation strain (ε_T,Sat_) required to achieve dynamic recovery of the microstructure are significantly higher than the strain necessary for necking formation (ε_T,Sat_ > ε_T,Neck_). This fact is supported by the decrease in the strain hardening exponent, which declines with increasing cold rolling deformation, as shown in Figure 12b.

## 4. Conclusions

Based on studies in the literature and our own experiments that were performed by cold rolling AISI 316 LN stainless steel to analyse stress–strain curves with a focus on mathematical description of the strain hardening rate and derivation of subsequent dependencies, the following conclusions can be drawn:

1. Measured values of engineering stress–strain curves resulting from static tensile tests were transformed into true stress–true strain curves and mathematically described by a rational polynomial function and the Holloman function with appropriate correlation;
2. Two points characterise the strain hardening rate: the initial strain hardening rate (θ_0_) and the maximal strain hardening rate (θ_Max_), These depend on cold rolling deformations and show an increase in their values. The dependence θ_Max_ = f(ε_Roll_) shows a local maximum at deformation ε_Roll_ = 30%. The curve θ_Max_ = f(d) exhibits a local maximum at the point where the diameter of the grain d = 77 μm, which also corresponds to ε_Roll_ = 30%;
3. Two areas describe a convex polyhedron characterised by uniform plastic deformations. One is characterised by the true strain curves and the other by the true stress curves. Both convex polyhedra describe areas of uniform plastic deformation, which are bounded by curves that represent initial stress–strain curves and stress–strain curves for necking. The values of the first convex polyhedron decrease and narrow with increasing cold rolling deformations, while the second one shows increasing values and narrowing with increasing cold rolling deformations;
4. The area of the stress convex polyhedron lies significantly above the curve describing the offset yield stress (σ_T,S_ > R_P0.2_). From this dependence, it follows that uniform plastic deformations do not occur near the offset yield stress;
5. The values of the true stresses and true strains indicate that the saturation stress and strain required to achieve dynamic recovery of the microstructure are significantly higher than the stress and strain needed to accomplish the necking (σ_T,Sat_ > σ_T,Neck_, ε_T,Sat_ > ε_T,Neck_). The graphical dependences show that, under the described cold deformation conditions, dynamic recovery of the microstructure is not possible.

## Figures and Tables

**Table 1:** Local chemical composition of AISI 316 LN [mass %].

C	Mn	Si	P	S	Cr	Ni	Mo	V	Ti	Nb	N	B
0.06	1.5	0.5	0.007	0.003	18.76	13.73	1.87	0.02	0.004	0.02	0.13	0.001

**Table 2:** Regression coefficients for Equation (6).

Rolling Deformation: ε_Roll_ [%]	Regression Coefficients			Correlation Index		
	A	B	C	D	E	
0	49,105	-	100	−263	358	0.85
10	209,459	−541,859	289	−1051	809	0.91
30	612,993	−3,299,235	568	−2905	-	0.9
50	510,209	-	290	8745	-	0.98

**Table 3:** Regression coefficients for Equation (7).

Rolling Deformation: ε_Roll_ [%]	Coefficients	
	**K**	**n**
0	1224	0.31
10	1229	0.22
30	1090	0.03
50	1112	0.0104

**Table 4:** Regression equations describing the relationship between strain hardening rate and true stress.

(θ_Max_, θ_0_)	Regression Equation	Correlation Index	Equation (No)
θ_Max_ = f(σ_T,U_)	$\theta_{Max}=-2.826\cdot 10^{7}+5.7186\cdot 10^{4}\cdot \sigma_{T,U}-28.32\cdot \sigma_{T,U}^{2}$	R^2^ = 0.99	(10)
θ_0_ = f(σ_T,U_)	$\theta_{0}=\left(236,906-532.97\cdot \sigma_{T,U}+0.3016\cdot \sigma_{T,U}^{2}\right)/\left(1-9.09\cdot 10^{-4}\cdot \sigma_{T,U}\right)$	R^2^ = 0.98	(11)
θ_Max_ = f(σ_T,S_)	$\theta_{Max}=\left(-34,669+80\cdot \sigma_{T,S}\right)/\left(1-0.0021\cdot \sigma_{S}+1.18\cdot 10^{-6}\cdot \sigma_{T,S}^{2}\right)$	R^2^ = 0.99	(12)
θ_0_ = f(σ_T,S_)	$\theta_{0}=6.815\cdot exp\left(0.01014\cdot \sigma_{T,S}\right)$	R^2^ = 0.99	(13)
θ_Max_ = f(σ_T,Peak_)	$\theta_{Max}=\left(-42,876+47.6\cdot \sigma_{Peak}\right)/\left(1-0.002024\cdot \sigma_{P}+1.032\cdot 10^{-6}\cdot \sigma_{Peak}^{2}\right)$	R^2^ = 0.99	(14)
θ_0_ = f(σ_T,Peak_)	$\theta_{0}=\left(501,599-1076.17\cdot \sigma_{T,Peak}+0.578\cdot \sigma_{T,Peak}^{2}\right)/\left(1-9.08\cdot 10^{-4}\cdot \sigma_{T,Peak}\right)$	R^2^ = 0.93	(15)
θ_Max_ = f(σ_T,DR_)	$\theta_{Max}=7690\cdot exp\left(0.0196\cdot \sigma_{DR}\right)$	R^2^ = 0.95	(16)
θ_0_ = f(σ_T,DR_)	$\theta_{0}=\left(8407.9-51.2\cdot \sigma_{T,DR}+0.07859\cdot \sigma_{T,DR}^{2}\right)/\left(1-4.495\cdot 10^{-3}\cdot \sigma_{T,DR}\right)$	R^2^ = 0.98	(17)

**Table 5:** Regression equations describing the relationship between true stress and coefficients.

(σ_T,Sat_, σ_T,DR_)	Regression Equation	Correlation Index	Equation (No)
σ_Sat_ = f(k)	$\sigma_{Sat}=1112+823.38/\left(k+0.6534\right)$	R^2^ = 0.97	(18)
σ_DR_ = f(k)	$\sigma_{DR}=\left(242.59+35.27\cdot k\right)/\left(1+0.1586\cdot k\right)$	R^2^ = 0.96	(19)
σ_Sat_ = f(n)	$\sigma_{Sat}=1124.5+8.246/\left(n-0.36\right)$	R^2^ = 0.98	(20)
σ_DR_ = f(n)	$\sigma_{DR}=306+42/\left(n-0.5\right)$	R^2^ = 0.99	(21)
σ_Sat_ = f(σ_DR)_	$\sigma_{Sat}=893.7\cdot exp\left(9.6\cdot 10^{-4}\cdot \sigma_{DR}\right)$	R^2^ = 0.98	(22)
n = f(k)	$n=(-0.239+1.97\cdot 10^{-3}\cdot k)/(1+0.251\cdot k)$	R^2^ = 0.99	(23)

## Data Availability

The original contributions presented in this study are included in the article. Further inquiries can be directed to the corresponding authors.
